# The impact of intermittent versus continuous exposure to EGFR tyrosine kinase inhibitor on selection of *EGFR* T790M-mutant drug-resistant clones in a lung cancer cell line carrying activating *EGFR* mutation

**DOI:** 10.18632/oncotarget.9703

**Published:** 2016-05-30

**Authors:** Youngjoo Lee, Yu-Ra Choi, Kyoung-Yeon Kim, Dong Hoon Shin

**Affiliations:** ^1^ Center for Lung Cancer, National Cancer Center, Goyang, Republic of Korea; ^2^ Lung Cancer Branch, Research Institute and Hospital, National Cancer Center, Goyang, Republic of Korea

**Keywords:** drug resistance, drug sensitivity, EGFR tyrosine kinase inhibitor, EGFR mutation, non-small-cell lung cancer

## Abstract

Drug-resistant cell lines are essential tools for investigating the mechanisms of resistance to molecular-targeted anti-cancer drugs. However, little is known about how to establish clinically relevant drug-resistant cell lines. Our study examined the impact of a drug-free period on the establishment of a cell line with clinically relevant resistance to molecular-targeted drugs. We used PC9 cells, a lung cancer cell line carrying *EGFR* mutation, because this is a validated target for EGFR tyrosine kinase inhibitors (TKI). PC9 cells were intermittently or continuously exposed to increasing concentrations of gefitinib (0.01 μM to 1.0 μM) and the emergence of the most common acquired resistance mutation in *EGFR*, T790M, was determined. T790M was detected at a 25-fold lower drug concentration in cells continuously exposed to gefitinib (PC9/GRc) than in cells intermittently exposed to gefitinib (PC9/GRi) (0.04 μM vs 1.0 μM, respectively). The mutation frequencies at those drug concentrations were 19.8% and 8.0% in PC9/GRc and PC9/GRi cells, respectively. After drug-free culture for 8 weeks, resistance to gefitinib decreased in the PC9/GRi cells but not in the PC9/GRc cells. In the PC9/GRc cells, the frequency of the T790M mutation was consistently about 20% from 0.04 μM to 1.0 μM of gefitinib. In the PC9/GRc cells, the T790M mutation was detected in all single-cell clones, at frequencies ranging from 7.0% to 37.0%, with a median of 19.5% (95% confidence interval, 17.3%–20.9%). In conclusion, compared with intermittent drug exposure, continuous exposure might select better minor drug-resistant clones when creating cell lines resistant to molecular-targeted drugs.

## INTRODUCTION

Recent technologic advances in identifying and targeting driving gene alterations has allowed highly effective and innovative treatments for human cancer to be developed, particularly molecular-targeted therapies. However, the emergence of drug resistance has become an inevitable problem even in the era of anti-cancer molecular-targeted therapies [1]. Thus, knowing the cause of resistance to molecular-targeted drugs has been more important to delay or overcome drug resistance. Both tissue samples and cell lines are conventionally used to investigate the mechanisms involved in drug resistance [1]. An artificially-established cell line is more commonly used in experiments to study the acquired drug resistance mechanisms because it is not feasible to collect tissue samples in many cases. However, an establishment of drug-resistant cell lines is mainly laborious, which takes from several months to years [2]. Furthermore, the clinical relevance of some drug-resistant cancer cell lines remains controversial [3, 4]. Thus, the appropriate strategy to efficiently make a clinically-relevant drug-resistant cell lines is much required to study the mechanism resistant to molecularly-targeted drugs.

There are several critical decisions to be made during the developing of drug resistant cell lines such as the choice of parent cell line, drug dose, and interval of drug treatment. Especially, drug treatment interval was seriously considered in earlier studies for cell lines resistant to conventional cytotoxic drugs like cisplatin and doxorubicin. Most patient receiving cytotoxic drugs are not continuously exposed to the drug, having the recovery period. Thus, previous studies elucidated that this treatment schedule might have an impact on the development of drug resistance. Some studies used an intermittent treatment method, in which the cells can periodically recover in drug-free media like the patient's real treatment schedule [5–7]. Other studies applied a continuous treatment method with stepwise drug dose escalation to establish more stable resistance [8–10]. The intermittent drug treatment was inferior in stability and strength of drug resistance whereas the continuous drug treatment was less clinically relevant even though it had high-level resistance [2]. On the other hand, for molecularly-targeted drug resistance, it is unknown about the impact of drug treatment schedule on making drug-resistant cell line.

This study examined the impact of a drug-free culture period on establishing a cell line with clinically relevant resistance to a molecular-targeted drug because previous studies using cytotoxic chemotherapy drugs showed the characteristics and strength of acquired drug resistance in an established cell line were influenced by a drug-free interval. Thus, the current study was designed to intermittently versus continuously expose a cancer cell line to a molecular-targeted drug and to determine the phenotypes and genotypes of the resistant cancer cells.

We used a lung cancer cell line carrying a mutation in the epidermal growth factor receptor gene (*EGFR*) because this is a validated target for EGFR tyrosine kinase inhibitors (EGFR-TKIs) [11–13]. EGFR-TKIs were the first molecular-targeted drugs to dramatically change the chemotherapeutic approach to lung cancer [14–16]. These drugs are particularly effective in lung cancers with activating *EGFR* mutations such as exon 19 deletion and exon 21 L858R mutation [11–13]. However, most cancers that have initial huge response to EGFR-TKIs eventually acquire drug resistance. Several mechanisms are responsible for acquired resistance to EGFR-TKIs, and the most common is the emergence of the T790M mutation in exon 20 of *EGFR* [17–19]. This secondary resistance mutation was detected in approximately 60% of rebiopsy samples obtained from patients with acquired resistance to EGFR-TKIs [17]. Furthermore, a preclinical study using established drug-resistant cell lines revealed the molecular mechanism of resistance induced by the T790M in *EGFR* [20]. Therefore, we measured the frequency of *EGFR* T790M in an *EGFR*-mutant lung cancer cell line resistant to EGFR-TKIs, which were established using two different drug-treatment regimens, in order to determine the relevance of these regimens to the emergence of clinically relevant drug resistance.

## RESULTS

### Generation of two drug-resistant cell lines

**Figure 1:**
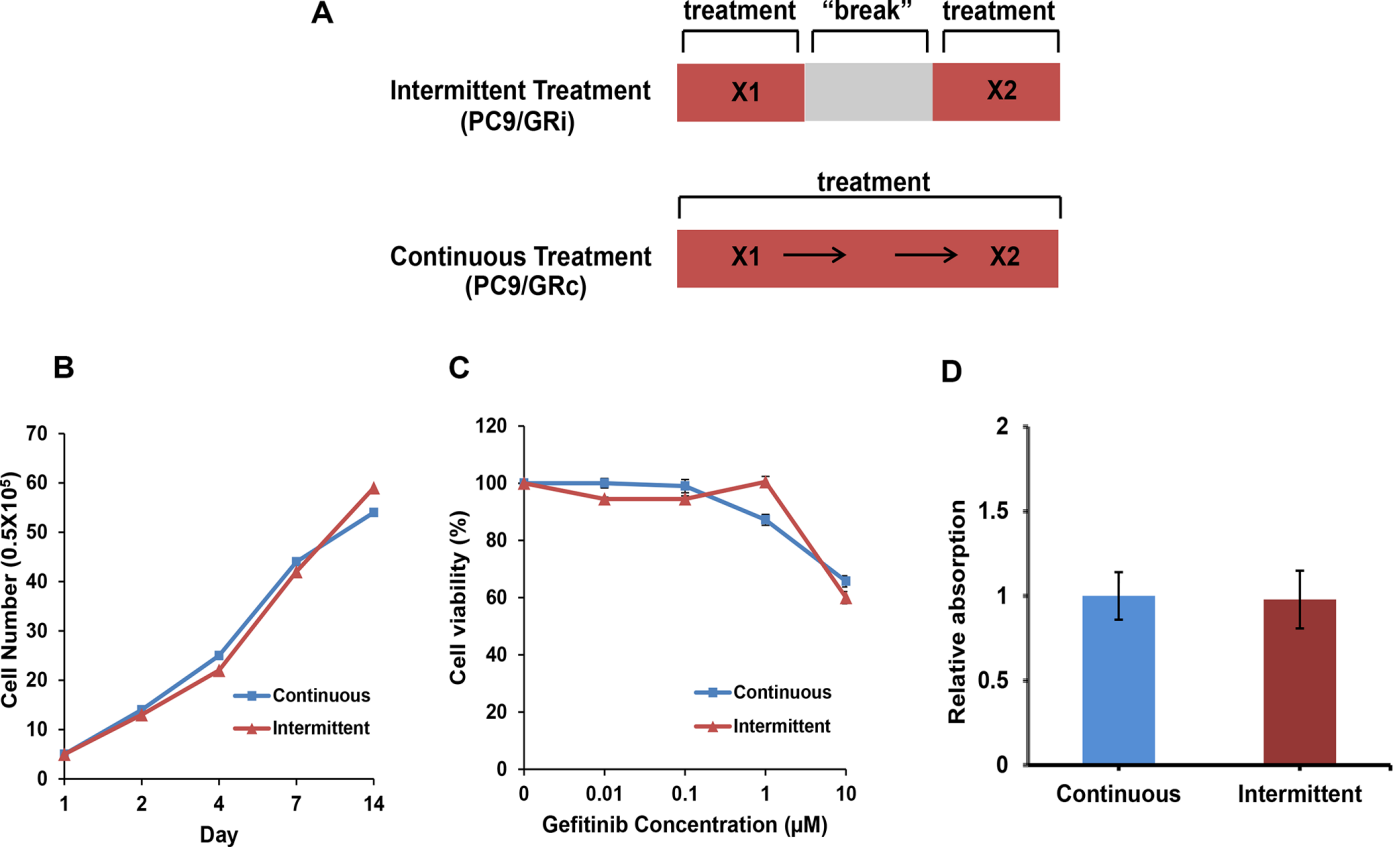
Characterization of two cell lines established with continuous or intermittent exposure to gefitinib (**A**) Schematic summary of two drug treatment methods (**B**) Both cell lines grew at similar rates over 2 weeks. (**C**) Cell viability assay was conducted after 72 h incubation on gefitinib. Both cell lines were resistant to gefitinib at similar concentrations (gefitinib IC_50_, 17.8 ± 1.2 μM in PC9/GRc and 15.8 ± 1.3 μM in PC9/GRi). (**D**) Migration activity was checked after 72 h incubation. There was no difference in migration activity between two cell lines. The graphs show the mean values of triplicate experiments and the error bars represent the standard deviations.

With continuous exposure to gefitinib (PC9/GRc cells), drug resistance was observed after 42 weeks, whereas with intermittent exposure (PC9/GRi cells), drug resistance was observed after 18 weeks (Figure 1A). The gross morphologies and growth rates of both resistant cell lines were similar (Figure 1B). Both cell lines also displayed similar sensitivities to gefitinib (gefitinib IC_50_, 17.8 μM in PC9/GRc and 15.8 μM in PC9/GRi) (Figure 1C). Additionally, there was no difference in migration activity between two cell lines (Figure 1D).

### Long-term stability of gefitinib resistance

**Figure 2:**
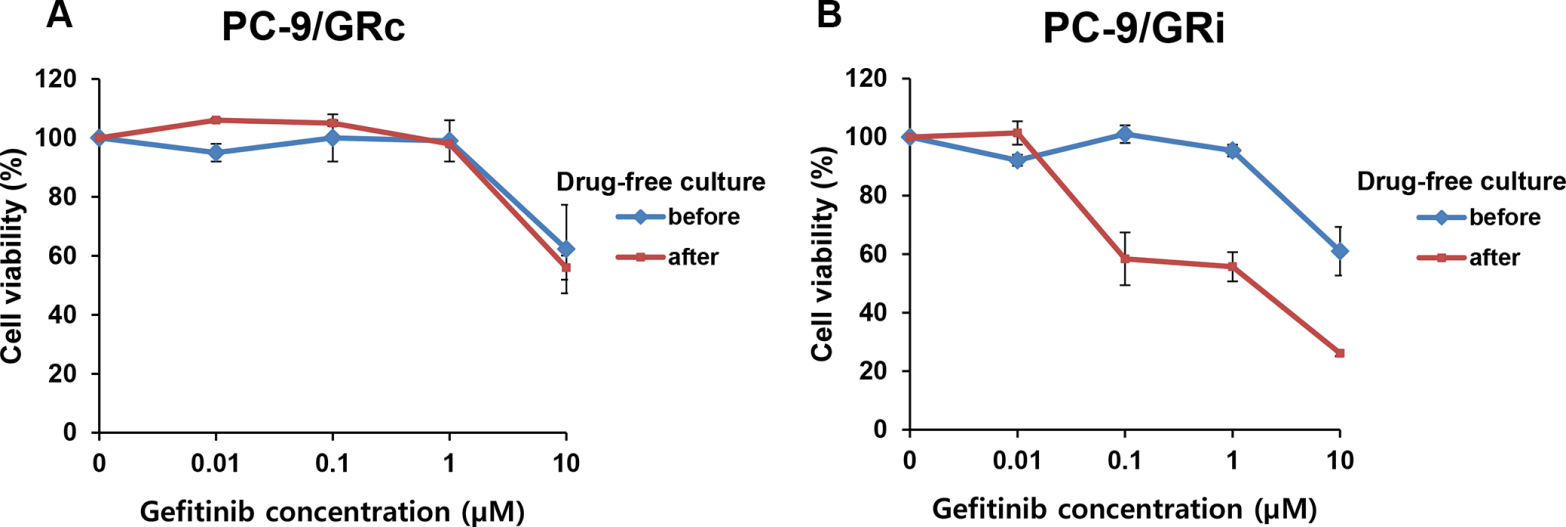
Long-term stability of gefitinib resistance in two cell lines established with continuous or intermittent exposure to gefitinib PC9/GRc and PC9/GRi cells were cultured in gefitinib-free medium for 8 weeks and their drug sensitivity was measured with an MTS cell proliferation assay after 72 h incubation on gefitinib. (**A**) The sensitivity of PC9/GRc cells to gefitinib was not significantly increased by drug-free culture (gefitinib IC_50_, 17.0 ± 1.2 μM and 13.5 ± 1.2 μM before and after drug-free culture, respectively). (**B**) The sensitivity of PC9/GRi cells to gefitinib increased significantly after drug-free culture (gefitinib IC_50_, 15.9 ± 1.2 μM and 1.04 ± 2.1 μM before and after drug-free culture, respectively). The graphs show the mean values of triplicate experiments and the error bars represent the standard deviations.

To assess the long-term stability of gefitinib resistance, the sensitivity of both cell lines to gefitinib was measured after drug-free culture for 8 weeks. The sensitivity of the PC9/GRc cells to gefitinib was not significantly different before and after drug-free culture (gefitinib IC_50_, 17.0 μM and 13.5 μM before and after drug-free culture, respectively) (Figure 2A). By contrast, the sensitivity of the PC9/GRi cells was significantly higher after drug-free culture compared with their sensitivity before drug-free culture lines (gefitinib IC_50_, 15.9 μM and 1.04 μM before and after drug-free culture, respectively) (Figure 2B).

### *EGFR* T790M mutation

**Figure 3:**
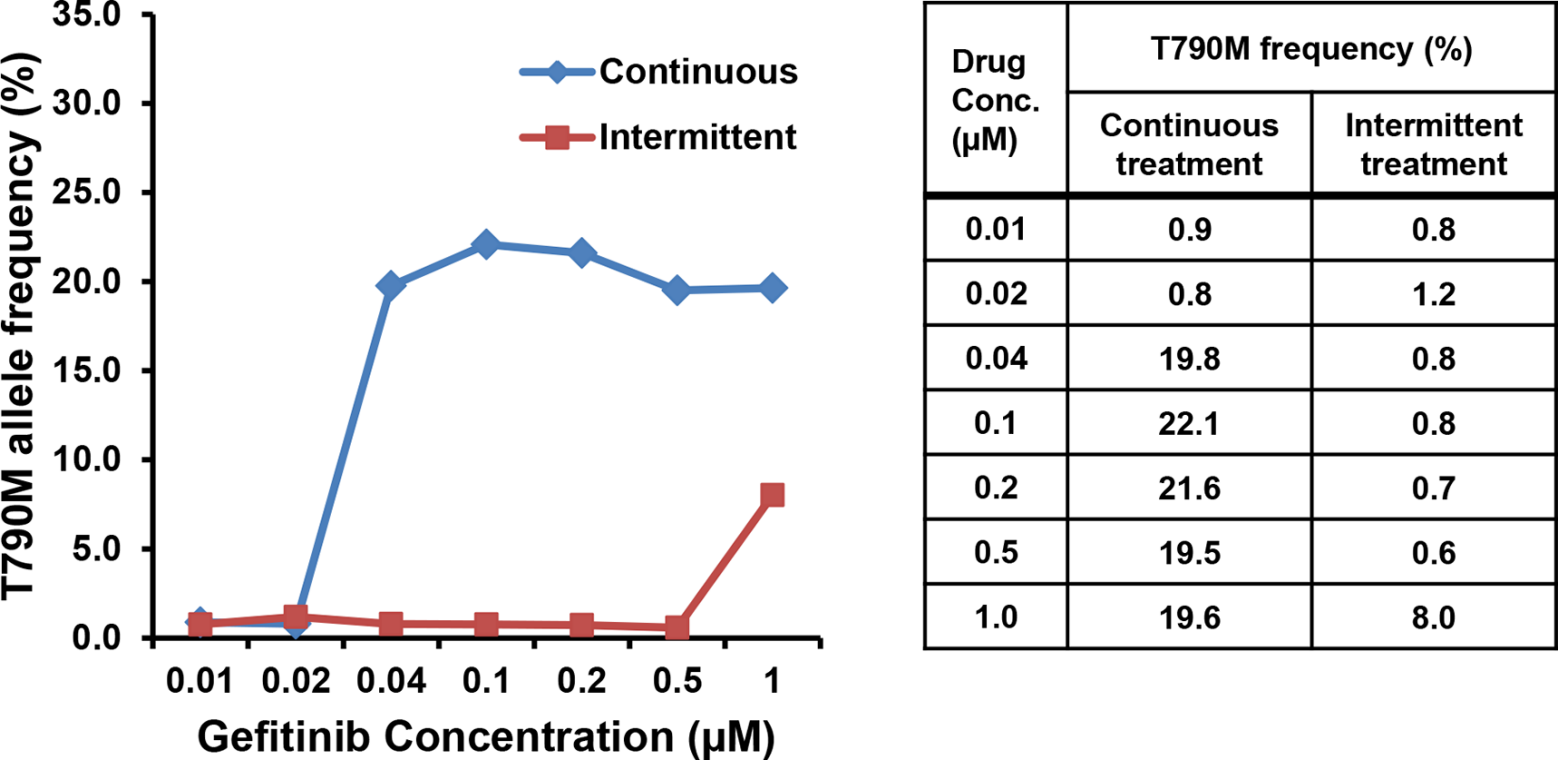
Differences in the frequency of the *EGFR* T790M mutation in the two cell lines established with continuous or intermittent exposure to gefitinib The frequency of T790M was measured semiquantitatively in both cell lines at each concentration of gefitinib with a mass spectrometry assay.

We next compared the frequency of the *EGFR* T790M mutation in the two drug-resistant cell lines. The Mass spectrometry (MS) assay was performed in both cell lines established at serially increasing concentrations of gefitinib (Figure 3). The lowest drug concentration at which T790M was detected was 0.04 μM in PC9/GRc cells and 1.0 μM in PC9/GRi cells. The mutant allele frequency at the lowest drug concentration at which T790M was detected was 19.8% in PC9/GRc cells and 8.0% in PC9/GRi cells.

**Figure 4:**
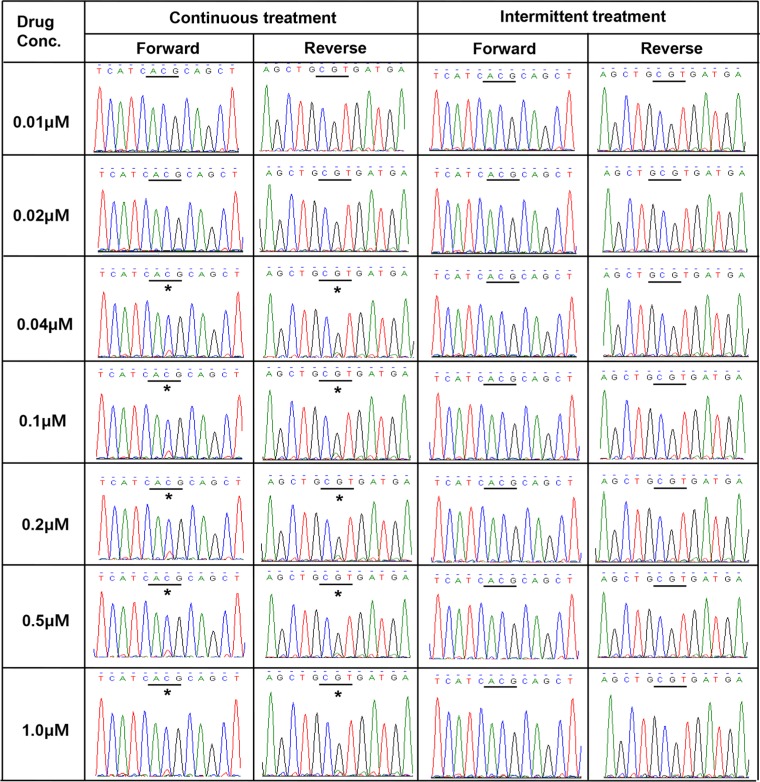
Direct sequencing chromatograms of *EGFR* exon 20 revealed the presence of T790M (*ACG→ATG) in PC9/GRc cells at gefitinib concentrations ranging from 0.04 μM to 1.0 μM, but not in PC9/GRi cells

In PC9/GRc cells, the allele frequency of T790M was approximately 20% at the lowest drug concentration at which T790M was detected and more, even at the highest drug concentration. The frequency of the T790M mutation in these cells did not increase as the drug concentration increased. These results are consistent with the results obtained with direct sequencing (Figure 4). Although the height of the mutant chromatogram peak was low, the peak corresponding to T790M was detected at each concentration from 0.04 μM to 1.0 μM in PC9/GRc cells, but was not detected in the PC9/GRi cells. The direct sequencing chromatogram also showed that the height of the T790M peak in the PC9/GRc cells did not increase as the drug concentration increased.

### Intra-cell-line T790M heterogeneity

**Figure 5:**
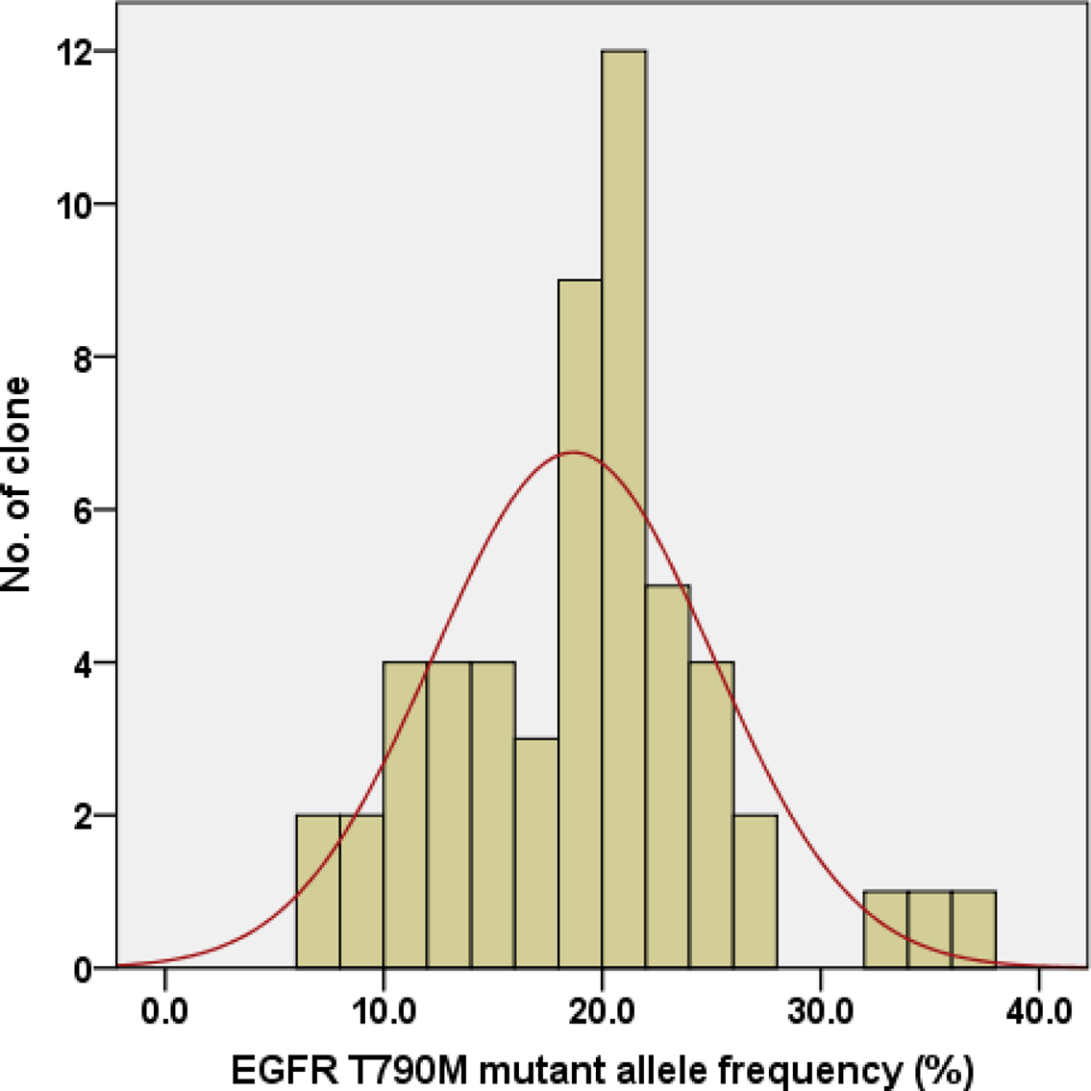
Distribution of the *EGFR* T790M mutant allele frequency in 54 single-cell clones isolated from PC9/GRc cells exposed to 1.0 μM gefitinib The median frequency was 19.5% (95% confidence interval 17.3%–20.9%).

**Figure 6:**
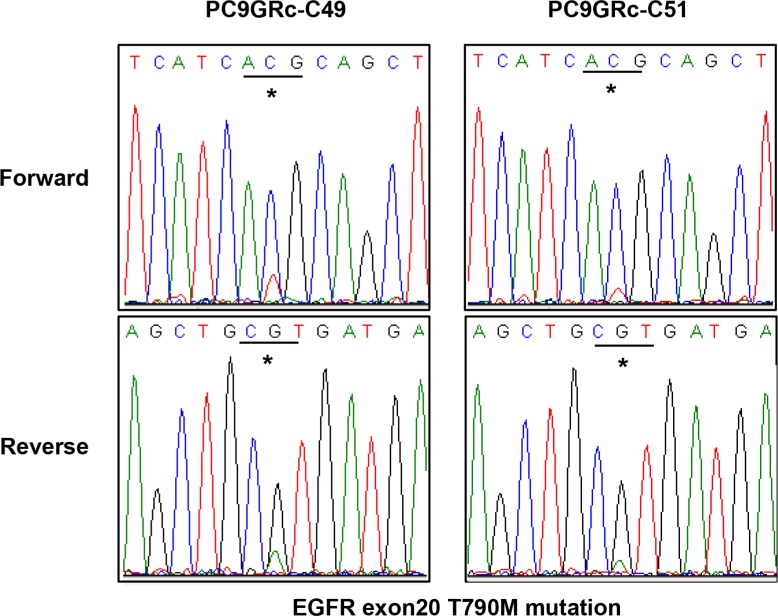
Direct sequencing chromatograms of *EGFR* exon 20 showed a difference in the peak at the site of the T790M mutation between two different single-cell clones derived from PC9/GRc cells

We examined whether there was intra-cell-line heterogeneity in the frequency of the *EGFR* T790M mutation in the PC9/GRc cells. Overall, 54 single-cell clones were isolated from PC9/GRc cells established with continuous exposure to 1.0 μM gefitinib. The MS assay for T790M was performed for each single-cell clone. T790M was detected in all single-cell clones, but the allele frequencies ranged considerably, from 7.0% to 37.0%, with a median of 19.5% (95% confidence interval 17.3%–20.9%; Figure 5). The frequency of T790M in PC9/GRc clone 51 was substantially lower than that in PC9/GRc clone 49, so these two representative single-cell clones displayed the heterogeneity of T790M. This heterogeneity was also detected with direct sequencing (Figure 6).

## DISCUSSION

This study tried to evaluate the effect of drug-free period in acquiring resistance to molecularly-targeted drugs in a cancer cell line. In this study, the intermittent drug exposure led to the establishment of cells with less stable drug resistance than the continuous drug exposure. Furthermore, the intermittent drug treatment was less effectively induced the emergence of the *EGFR* T790M mutation which is the most common resistance mechanism found in *EGFR*-mutant lung cancer patients treated by EGFR-TKI, compared with the continuous drug treatment. We could not detect *EGFR* T790M mutation in the PC9/GRi cells by a standard direct sequencing method having a detection limit of about 20%, even when they had the highest-level resistance to the drug. These findings are consistent with those of another study in which the researchers used intermittent drug exposure to establish a lung cancer cell line resistant to an EGFR-TKI [21]. Rho *et al*. generated resistant PC9 cells by exposing them to gefitinib or erlotinib for 48 h, maintaining them in drug-free medium, and then re-exposing them to increasing concentrations of the drug to a final concentration of 1.0 μM. They were unable to detect T790M in the drug-resistant cell lines with direct sequencing, although when they used the more sensitive pyrosequencing method, the mutation frequency was 13%~14%. These results suggest that when the drug selection pressure gets removed, the residual drug-sensitive clones can be restored, expand, and then suppressed the expansion of other drug-resistant clones. Taken together, these findings indicate that continuous drug exposure offers an advantage over intermittent drug exposure in terms of long stability and mimicking the molecular change observed in the patients.

Several studies support our finding that continuous exposure to a molecular-targeted drug selects better drug-resistant tumor cells than intermittent exposure [22, 23]. For example, using a mathematical cancer model and *EGFR*-mutant lung cancer cell lines, Chmielecki *et al.* showed that a high–dose pulse dosing combined with a continuous low dosing of EGFR-TKI delayed the emergence of T790M-mediated resistance compared with its emergence in cells treated with a continuous standard dosing [22]. This research group has implemented a phase I clinical trial based on these preclinical data. The results of their studies should help us improve the clinical efficacy of EGFR-TKIs in the treatment of *EGFR*-mutant lung cancers by delaying the emergence of drug resistance. Additionally, Thakur *et al*. reported that drug-resistant cells showed continuous dependence on a B-RAF inhibitor in *BRAF*-mutated melanoma cell lines and that an intermittent dosing strategy delayed the onset of drug resistance in a xenograft tumor model [23]. Based on these preclinical data, a clinical trial has been commenced to compare the efficacy of intermittent dosing and continuous dosing with a B-RAF inhibitor and a MEK inhibitor in patients with *BRAF*-mutant melanoma.

Interestingly, there is an apparent drug concentration at which the expansion of drug-resistant clones plateaus, because the frequency of T790M remained constant once the drug concentration exceeded this value. This indicates that the strength of resistance does not correlate positively with the frequency of the resistance mutation. Unfortunately, we could not determine the clinical relevance of these results because there are no clinical data on the frequency of the EGFR T790M mutation in drug-resistant tumor tissues. However, if we knew the threshold concentration at which drug resistance is expected to develop, we could save time and labor in producing cell lines with resistance to molecular-targeted drug that mimic the molecular characteristics of human tumors. In addition, we observed individual clones within PC9 cell population acquiring resistance to gefitinib have a different allele frequency of T790M mutation. This finding may be explained by innate clonal heterogeneity of the tumor. Several clinical studies suggested the presence of clone with T790M-resistant mutation before exposure to EGFR-TKI in *EGFR*-mutant lung cancer [24–26]. The recent preclinical study by Hata A.N. *et al* directly detected the pre-existing *EGFR*-T790M clones in PC9 cell carrying 8 to 10 copies of *EGFR* gene [27]. Therefore, it is necessary to consider clonal diversity in the phenotypes and genotypes of single-cell drug-resistant clones when they are used to examine the mechanisms of drug resistance.

In conclusion, cancer cell lines are an important tool in studying the mechanisms underlying drug resistance in human cancer. Our findings indicate that the mechanisms responsible for the resistant phenotype of cell lines can vary according to the conditions used to establish the cells, especially the use of intermittent or continuous drug exposure regimens. Compared with intermittent drug exposure, continuous drug exposure might select better minor resistant clones when creating cell lines resistant to molecular-targeted drugs.

## MATERIALS AND METHODS

### Cell line and reagents

PC9 cells, a human lung cancer cell line carrying a deletion in exon 19 (DelE746A750) of *EGFR*, were purchased from RIKEN BioResource Center Cell Bank (Ibaraki, Japan). The cells were maintained in RPMI-1640 supplemented with 10% fetal bovine serum. An EGFR-TKI, gefitinib (Iressa^®^), was purchased from LC Laboratories (Woburn, MA, USA).

### Establishment of gefitinib-resistant cell lines

The gefitinib-resistant cell line (PC9/GR) was generated by continuously exposing PC9 cells to increasing concentrations of gefitinib. Starting at a concentration of 0.01 μM, the exposure dose was doubled until it reached a final concentration of 1.0 μM. We used two different drug treatment regimens, intermittent and continuous exposure (Figure 1A). Cells in the intermittent treatment group (PC9/GRi) were exposed to gefitinib in culture medium for 72 h, washed, and then cultured in gefitinib-free medium until their growth rate was similar to that of the parental cells. The cells in the continuous treatment group (PC9/GRc) were continuously exposed to gefitinib at a given concentration and the medium was not changed to drug-free medium at any time. When the growth rate of these cells was equal to that of the parental cells, they were exposed to increasing concentrations of gefitinib. The drug-resistant phenotypes of both groups of cells were confirmed with a cell viability assay. An aliquot of cells was stored before each increase in the dose of gefitinib.

### Cell viability assay

The cells were cultured in gefitinib-free medium for ≥ 1 week before testing. The cells were then seeded at a density of 4 × 10^3^ cells/well in 96-well plates. After 24 h, the cells were exposed to different concentrations of gefitinib and were incubated for 72 h. The cells were then washed with phosphate-buffered saline and the cell viability was measured with the CellTiter 96^®^ AQueous One Solution Cell Proliferation Assay (Promega, Madison, WI, USA), according to the manufacturer's instructions. We estimated IC_50_ for gefitnib using GraphPad software.

### Direct sequencing

Genomic DNA was extracted from the cells with a DNeasy Tissue Kit (Qiagen, Valencia, CA, USA), according to the manufacturer's instructions. Polymerase chain reaction (PCR) amplification was performed with 5 μl of the extracted genomic DNA, 1 U of *Taq* DNA polymerase, 0.25 mM each dNTP, 10 mM Tris-HCl, 40 mM KCl, 1.5 mM MgCl_2_, and 20 pmol of the primers in a final volume of 20 μl. The following primers were used to amplify exon 20 of *EGFR*: 5′-CCATGAGTACGTATTTTGAAAC- TC-3′(forward) and 5′-CATATCCCCATGGCAAACTCTTGC-3′ (reverse). The PCR cycling parameters were 95°C for 5 min, 40 cycles at 95°C for 30 s, 55°C for 30 s, and 72°C for 30 s, followed by a final step at 72°C for 10 min. After the PCR products were purified, they were directly sequenced with the MegaBACE DNA Analysis System (Amersham Biosciences, Sunnyvale, CA, USA), with a standard published protocol.

### Mass spectrometry (MS) assay

The EGFR T790M mutation was detected in the cells with matrix-assisted laser desorption/ionization time-of-flight MS using a standard protocol, on the MassARRAY System (Sequenom, San Diego, CA, USA). The mutant signal frequency was calculated as follows: mutant signal frequency (%) = (mutant peak height)/(mutant peak height + wild-type peak height) × 100.

### Single-cell clone assay

Individual clones were established from the resistant cell lines. Briefly, 4 × 10^3^ drug-resistant cells were serially diluted and then incubated in 96-well plates. Each well was checked to identify those that contained a single colony. These colonies were picked from the wells and subcultured in larger vessels.

### Cell migration assay

The migration assay was performed using transwell plates (Corning Costar, Cambridge, MA, USA) that were 6.5 mm in diameter with 8 μm pore filter. The RPMI medium containing 10% FBS was inserted into the lower chamber. Cells were plated in 1.5 ml of serum-free RPMI per filter with a cell density of 5 × 10^4^. Cells were allowed to migrate in 5% CO_2_ at 37°C for 72 hr and were subsequently fixed by immersion of the filters in methanol at room temperature for 15 min. Filters were washed and stained in 0.2% crystal violet in a 20% methanol/water solution for 10 min. Cells were removed from the upper chamber with a cotton swab. The absorbance at 590 nm of each filters was measured with an enzyme-labeling measuring instrument (Gene Company Limited).
